# Accelerated long‐term forgetting is a BACE1 inhibitor‐reversible incipient cognitive phenotype in Alzheimer’s disease model mice

**DOI:** 10.1002/npr2.12174

**Published:** 2021-03-22

**Authors:** Masuo Ohno

**Affiliations:** ^1^ Center for Dementia Research Nathan Kline Institute Orangeburg NY USA; ^2^ Department of Psychiatry New York University School of Medicine New York NY USA

**Keywords:** 5XFAD, accelerated long‐term forgetting, Alzheimer's disease, BACE1 inhibitor, presymptomatic therapy

## Abstract

**Aim:**

After the continued failure of β‐secretase (BACE1) inhibitor clinical trials in prodromal as well as mild‐to‐moderate Alzheimer's disease (AD), they are shifting to further earlier or asymptomatic stages. The aim of this study is to explore a cognitive paradigm that allows us to more sensitively detect beneficial effects of BACE1 inhibitors in presymptomatic AD.

**Methods:**

GRL‐8234 (33.4 mg/kg, ip), a small‐molecule BACE1 inhibitor, was administered once daily for 28 days to the 5XAFD transgenic mouse model of AD. The contextual fear conditioning was used to evaluate the effects of GRL‐8234 on memory deficits in 5XFAD mice at different ages.

**Results:**

Chronic administration of GRL‐8234 to 5XFAD mice rescued their contextual memory deficits, when tested 1 day after training at 6‐8 months but not at 12 months of age. Importantly, 4‐month‐old 5XFAD mice retain the ability to form contextual memory equivalent to wild‐type controls, demonstrating that the standard method of 1‐day memory assessment is not suitable for evaluating BACE1 inhibitor efficacy in ameliorating cognitive declines during earlier disease stages. Despite normal contextual memory formation, young 5XFAD mice showed faster forgetting when a longer delay (28 days) intervened between training and memory testing. Notably, GRL‐8234 administered to 4‐month‐old 5XFAD mice during the 28‐day delay reversed accelerated long‐term forgetting almost completely back to wild‐type control levels.

**Conclusion:**

The results provide experimental evidence that accelerated long‐term forgetting represents more sensitive memory testing that can help evaluate BACE1 inhibitor therapy in presymptomatic AD populations.

## Abstract 2

Despite intact 1‐day contextual memory, 4‐month‐old 5XFAD mice showed faster forgetting when a longer delay (28 days) intervened between training and memory testing. Notably, accelerated long‐term forgetting was reversed by the BACE1 inhibitor GRL‐8234 administered during the 28‐day delay. Therefore, accelerated long‐term forgetting represents a sensitive paradigm that can detect subtle cognitive decline and help evaluate early BACE1 interventions during presymptomatic stages of Alzheimer's disease.

## Graphical abstract

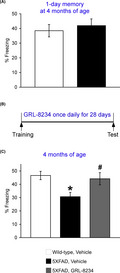

## 1 INTRODUCTION

β‐Site amyloid precursor protein (APP)‐cleaving enzyme 1 (BACE1 or β‐secretase) is a prime drug target for Alzheimer's disease (AD),^1^, ^2^ since this enzyme is responsible for initiating the generation of amyloid‐β (Aβ) that triggers a pathogenic cascade ultimately leading to neuron death and memory deficits.^3^ However, BACE1 inhibitor clinical trials have been failing to show beneficial cognitive outcomes first in patients with mild‐to‐moderate AD^4^, ^5^ and more recently in those with early stages of mild AD or amnesic mild cognitive impairment (MCI) due to AD (prodromal AD).^6^, ^7^, ^8^ Clinical studies are shifting to target further earlier stages such as secondary prevention (presymptomatic populations) and primary prevention (before amyloid build‐up preceding symptoms by ~ 15 years^9^) of AD. These efforts are, undoubtedly, indispensable for determining the disease stage to start successful BACE1 inhibitor interventions for clinical efficacy, while evaluating the cognitive benefits (if any) in asymptomatic subjects at a risk of developing AD is challenging.

Remarkably, accelerated long‐term forgetting is emerging as a new cognitive measure sensitive to presymptomatic changes in familial AD individuals^10^, ^11^ and ApoE ε4 carriers^12^, ^13^ who are still normal in standard or short‐term memory testing. Consistent with these clinical observations, very young AD transgenic mice including our 5XFAD model retain the ability to learn a contextual or spatial memory task and intact hippocampal long‐term potentiation (LTP), while they show faster forgetting after a long delay concomitant with cortical dysfunction.^14^, ^15^ In this study, I tested whether GRL‐8234, a well‐established BACE1 inhibitor,^16^, ^17^ can reverse accelerated long‐term forgetting in young 5XFAD mice, of which CA1 LTP or contextual memory formation is not yet impaired.^14^ The results have important implications for designing more sensitive memory paradigms to evaluate BACE1 inhibitor therapy in treating individuals during preclinical stages of AD.

## 2 METHODS

### 2.1 Animals

5XFAD mice co‐overexpress five familial AD mutant forms of human APP (Swedish, Florida, and London mutations) and presenilin 1 (M146L and L286V) transgenes under the neuron‐specific Thy‐1 promoter.^18^, ^19^ 5XFAD line (Tg6799) was maintained by crossing hemizygous transgenic mice with C57Bl/6 breeders (Taconic, Hudson). 5XFAD hemizygotes were used for the experiment with wild‐type littermate mice served as controls. Genotyping was performed by PCR analysis of tail DNA. Our previous study showed no sex difference in cerebral Aβ levels in 5XFAD mice except for young age (≤3 months);^19^ therefore, both males and females were used to examine the effects of GRL‐8234 in this model at ≥ 4 months of age. All animal procedures were approved by the Nathan Kline Institute Animal Care and Use Committee.

### 2.2 Drug treatments

The potent and selective BACE1 inhibitor GRL‐8234 (Dr Tang, Oklahoma Medical Research Foundation) was dissolved in 50:50 mixture of 0.5% polyethylene glycol 300 and 5% glucose, as described.^16^, ^17^ 5XFAD transgenic mice at different ages received intraperitoneal injections of 33.4 mg/kg GRL‐8234 once daily for 28 days. Age‐matched wild‐type mice were treated with vehicle as controls. The dosage of 33.4 mg/kg was chosen on the basis of a previous study that demonstrated that systemic GRL‐8234 enters brain (16% over a 24‐hours period) and a single daily dose is sufficient to maintain significant inhibition of Aβ production (~50% in basal interstitial Aβ levels) in Tg2576 mice.^16^ Our study also showed that chronic administration of 33.4 mg/kg GRL‐8234 to 5XFAD mice can reduce cerebral Aβ levels concomitant with ~ 50% inhibition of the β‐cleavage of APP, as measured by its direct β‐metabolites, regardless of age.^17^

### 2.3 Contextual fear conditioning

Contextual fear conditioning was conducted as described previously.^14^ In brief, mice were placed in the conditioning chamber for 3 minutes and then received a footshock (0.8 mA, 2 seconds) during training. After the shock delivery, mice were left in the chamber for another 30 seconds. Contextual fear memory was evaluated by scoring freezing behavior (the absence of all movement except for that needed for breathing) for 3 minutes when the mice were placed back into the same conditioning chamber 1 day or 28 days after training. The automated FreezeFrame system (Coulbourn Instruments, Allentown) was used to score the amount of freezing.

### 2.4 Data analysis

The significance of differences between the groups was determined by a one‐way ANOVA, and post hoc Fisher's PLSD tests were performed when appropriate. Data were presented as mean ± SEM, and the level of significance was set at *P* < .05.

## 3 RESULTS

### 3.1 Effects of GRL‐8234 on deficient 1‐day memory in older 5XFAD mice

**FIGURE 1:**
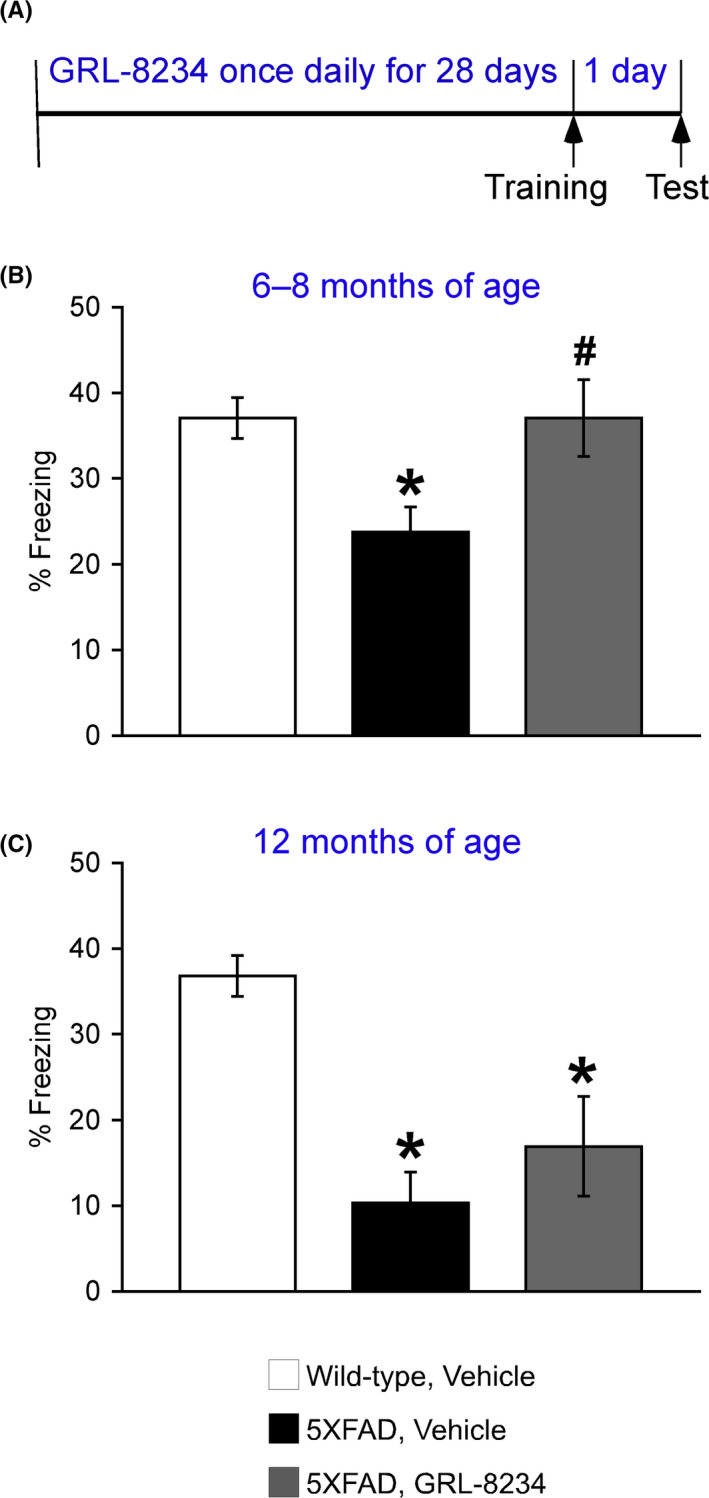
Effects of the BACE1 inhibitor GRL‐8234 on the impairment of 1‐day contextual memory in 5XFAD mice. A, Experimental protocols for GRL‐8234 treatments and the contextual fear conditioning. B, C, Treatments with 33.4 mg/kg GRL‐8234 for 28 days before training reversed 1‐day memory deficits in 5XFAD mice at 6‐8 months but not at 12 months of age (n = 10‐23 mice per group). Data are presented as mean ± SEM. One‐way ANOVA followed by Fisher's PLSD test; * *P* < .05 (vs wild‐type, vehicle), # *P* < .05 (vs 5XFAD, vehicle)

5XFAD mice begin to develop visible Aβ deposition as early as 2 months of age and exhibit significant memory impairments on a battery of hippocampus‐dependent tasks (incl. the contextual fear conditioning) at ~ 6 months concomitant with moderate Aβ accumulation and the onset of Schaffer collateral‐CA1 synaptic dysfunction.^14^, ^18^, ^20^, ^21^ Therefore, I first compared the effects of GR‐8234 on memory deficits in 5XFAD mice at 6‐8 and 12 months of age after they received 33.4 mg/kg GRL‐8234 once daily for 28 days (Figure 1A). Consistent with our previous results, vehicle‐treated 5XFAD control mice showed significantly lower levels of freezing 1 day after training in both age groups, as compared with the respective wild‐type controls (*P* < .05). GRL‐8234 significantly increased contextual freezing in 5XFAD mice at 6‐8 months of age (*P* < .05), restoring the impaired 1‐day memory completely back to wild‐type levels (Figure 1B). In contrast, levels of freezing were indistinguishable between GRL‐8234‐ and vehicle‐treated 5XFAD mice at 12 months of age (Figure 1C). Together, the results indicate that chronic treatment with GRL‐8234 reversed deficits in 1‐day contextual memory in 5XFAD mice at 6‐8 months but not at 12 months of age.

### 3.2 Effects of GRL‐8234 on accelerated long‐term forgetting in younger 5XFAD mice

**FIGURE 2:**
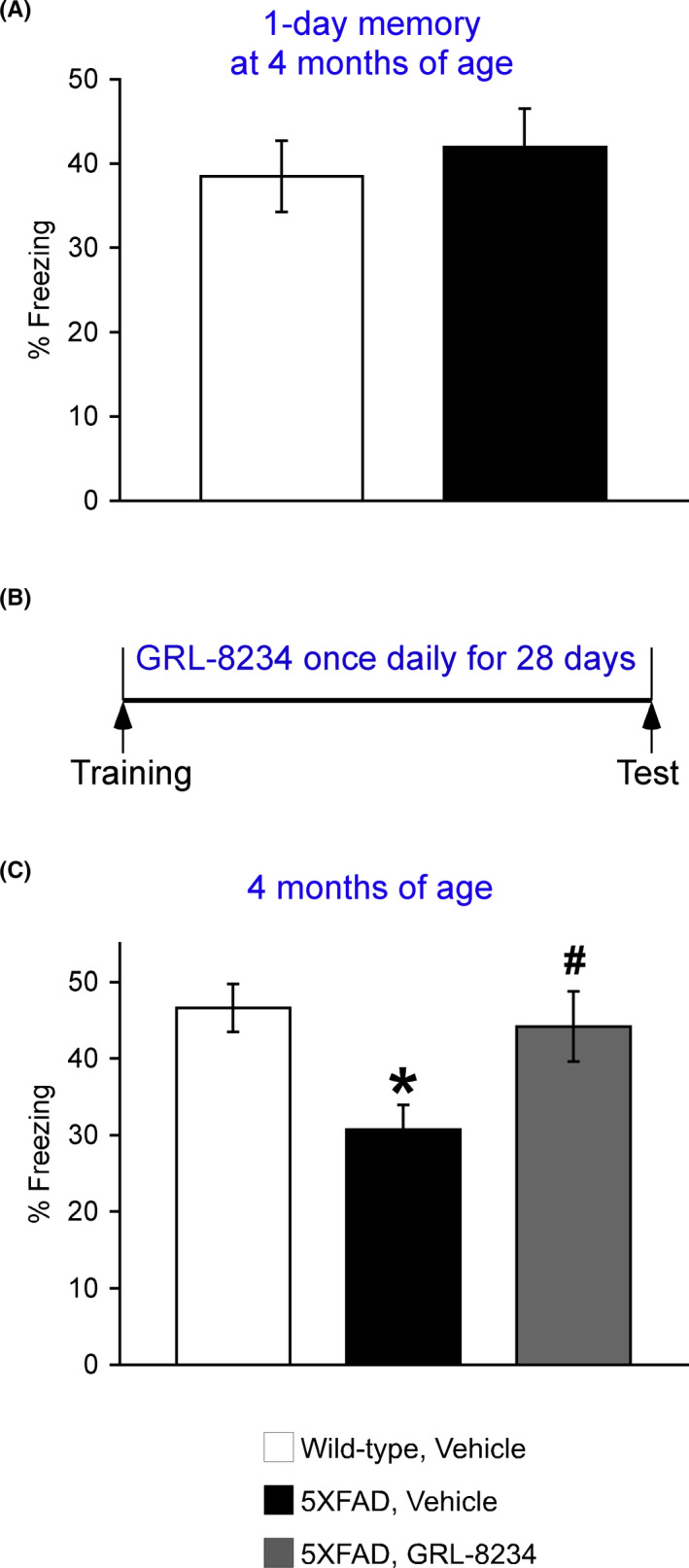
Effects of the BACE1 inhibitor GRL‐8234 on accelerated long‐term forgetting in 5XFAD mice. A, Contextual memory was not impaired in 5XFAD mice at 4 months of age when tested 1 day after training (n = 8 mice per group). B, Experimental protocols for GRL‐8234 treatments and testing of long‐term forgetting in the contextual fear conditioning. C, Treatments with 33.4 mg/kg GRL‐8234 during a 28‐day delay after training reversed accelerated long‐term forgetting in 4‐month‐old 5XFAD mice. Data are presented as mean ± SEM (n = 13‐29 mice per group). One‐way ANOVA followed by Fisher's PLSD test; * *P* < .05 (vs wild‐type, vehicle), # *P* < .05 (vs 5XFAD, vehicle)

Four‐month‐old 5XFAD mice were next tested in the contextual fear conditioning. However, the standard method of 1‐day memory assessment was unable to detect a difference between 5XFAD and wild‐type controls (Figure 2A), precluding this paradigm from evaluating positive drug effects (if any) at the younger age. Therefore, we explored whether GRL‐8234 could affect a long‐term forgetting process when a longer delay (28 days) intervened between training and memory testing (Figure 2B). Notably, young 5XFAD control mice showed faster forgetting compared with wild‐type mice (*P* < .05), while GRL‐8234 administered during the 28‐day delay reversed accelerated long‐term forgetting in 5XFAD mice almost completely back to wild‐type control levels (*P* < .05) (Figure 2C). The results suggest that testing memory after a longer delay may help more sensitively detect subtle declines and evaluate the cognitive efficacy of BACE1 inhibitors at an incipient stage of AD.

## 4 DISCUSSION

Consistent with our previous findings in the spontaneous alternation Y‐maze task,^17^ GRL‐8234 better worked in rescuing deficient contextual memory formation in 5XFAD mice if applied in a preventive style (ie, before the onset of hippocampal LTP and mnemonic declines at 6 months of age) rather than in a therapeutic mode (ie, after pronounced Aβ deposition and memory deficits occurred at 12 months). Our study supports the idea that addressing AD at earlier stages should increase the likelihood of BACE1 inhibitor drugs being effective. Further study is required to determine whether the benefits of early BACE1 interventions may last until later disease stages.

More importantly, the standard procedure of 1‐day memory assessment was not sensitive enough for detecting subtle declines in the contextual fear conditioning in 4‐month‐old 5XFAD mice, while a longer delay (28 days) intervening between training and memory testing revealed accelerated long‐term forgetting that was reversible with chronic administration of GRL‐8234. Consistent with our findings in young 5XFAD mice,^14^ pre‐pathological PDAPP transgenic mice show cortical glucose‐uptake deficiency and faster forgetting after 7‐day delay despite normal spatial memory formation in the water maze.^15^ Accelerated long‐term forgetting, in which memory impairment becomes apparent over 7‐day to 3‐month retention intervals, is also identified as an early presymptomatic feature of subtle cognitive dysfunction in familial AD individuals^10^, ^11^ and ApoE ε4 carriers^12^, ^13^ whose performance on standard short‐term memory testing is still intact. Mechanistically, it remains unclear whether faster forgetting after a long delay that follows intact memory encoding may reflect forgetting of hippocampus‐dependent memory or the impairment of systems memory consolidation, which takes place in cortical networks through hippocampal‐cortical interactions.^22^, ^23^ Interestingly, cortical areas including the anterior cingulate responsible for remote memory consolidation^22^, ^23^ show highest regional Aβ load closely related to local atrophy in populations with subjective cognitive impairment before diagnosis.^24^ Collectively, the findings strongly suggest that faster forgetting that is detectable after a prolonged delay represents a sensitive cognitive measure useful for evaluating BACE1 inhibitor therapy in presymptomatic AD trials.

A growing number of BACE1 substrates besides APP uncover new physiological roles of this protease,^25^, ^26^ implying a need for the successful balance between tolerable mechanism‐based side effects and sufficient Aβ reductions for efficacy. Concerning the failure of recent clinical trials, it should be noted that BACE1 inhibitor drugs showed dose‐dependent cognitive worsening (rather than improvement) in prodromal AD populations.^6^, ^7^, ^8^ This was observed for multiple BACE1 inhibitors at the highest dose, most likely indicative of side effects associated with excessive BACE1 inhibition rather than compound‐specific effects. The current clinical dosage achieving up to 90% Aβ reductions seems far higher than needed, given that an APP mutation (A673T) that lowers Aβ in plasma by only 28% can protect against AD and age‐related cognitive decline.^27^, ^28^ In fact, chronic administration of high‐dosage BACE1 inhibitors to normal adult mice has been reported to interfere with structural (spine formation) and functional (LTP) synaptic plasticity in the hippocampal CA1, as observed in BACE1^−/−^ knockouts.^26^, ^29^, ^30^ Importantly, the dose of 33.4 mg/kg GRL‐8234 applied in this study induces ~ 50% inhibition of the β‐cleavage of APP and moderate Aβ reduction in 5XFAD mice without adverse cognitive effects^17^ in accordance with BACE1^+/–^ reduction or the lower dose of inhibitors devoid of synaptic side effect.^1^, ^26^ Taken together, the results support the idea that future clinical trials should be optimized by initiating BACE1 inhibitor interventions at the earliest preclinical stages of AD, targeting safe levels of reduction in β‐secretase activity (50% or less), and applying more sensitive memory paradigms such as accelerated long‐term forgetting.

## CONFLICT OF INTEREST

The author declares no conflict of interest.

## AUTHOR CONTRIBUTION

MO designed the research, performed the experiments, analyzed the data, and wrote the manuscript.

## ANIMAL STUDIES

All animal experiments were approved by the Animal Care and Use Committee of Nathan Kline Institute.

## Supporting information

Table S1

## Data Availability

The data that support the findings of this study are available in the Supporting Information of this article.
